# The Power and the Promise of Cell Reprogramming: Personalized Autologous Body Organ and Cell Transplantation

**DOI:** 10.3390/jcm3020373

**Published:** 2014-04-04

**Authors:** Ana Belen Alvarez Palomo, Michaela Lucas, Rodney J. Dilley, Samuel McLenachan, Fred Kuanfu Chen, Jordi Requena, Marti Farrera Sal, Andrew Lucas, Inaki Alvarez, Dolores Jaraquemada, Michael J. Edel

**Affiliations:** 1 Control of Pluripotency Laboratory, Department of Physiological Sciences I, Faculty of Medicine, University of Barcelona, Hospital Clinic, Casanova 143, Barcelona 08036, Spain; E-Mails: b.alvarez@ub.edu (A.B.A.P.); joreos@hotmail.com (J.R.); marti.farrera@gmail.com (M.F.S.); 2 School of Medicine and Pharmacology, University of Western Australia, Crawley, Perth, WA 6009, Australia; E-Mail: michaela.lucas@health.wa.gov.au; 3 Pathwest, Nedlands, WA 6009, Australia; 4 Ear Sciences Centre, School of Surgery, University of Western Australia, Nedlands, WA 6009, Australia; E-Mail: rodney.dilley@uwa.edu.au; 5 Centre for Ophthalmology and Visual Science (Lions Eye Institute), University of Western Australia, Perth, WA 6009, Australia; E-Mails: samuelmclenachan@lei.org.au (S.M.); fredchen@lei.org.au (F.K.C.); 6 Institute for Immunology and Infectious Diseases, Murdoch University, Murdoch, Perth, WA 6150, Australia; E-Mail: a.lucas@iiid.com.au; 7 Immunology Unit, Department of Cell Biology, Physiology and Immunology and Institute of Biotechnology and Biomedicine, Autonomous University of Barcelona, Bellaterra 08193, Spain; E-Mails: inaki.alvarez@uab.es (I.A.); dolores.jaraquemada@uab.es (D.J.); 8 Senior Research Fellow at the University of Sydney Medical School, Faculty of Medicine, Westmead Children’s Hospital, Division of Pediatrics and Child Health, Sydney, NSW 2145, Australia

**Keywords:** direct cell reprogramming, iPSC, autologous, immune response, organ bioengineering

## Abstract

Reprogramming somatic cells to induced pluripotent stem cells (iPSCs) or direct reprogramming to desired cell types are powerful and new *in vitro* methods for the study of human disease, cell replacement therapy, and drug development. Both methods to reprogram cells are unconstrained by the ethical and social questions raised by embryonic stem cells. iPSC technology promises to enable personalized autologous cell therapy and has the potential to revolutionize cell replacement therapy and regenerative medicine. Potential applications of iPSC technology are rapidly increasing in ambition from discrete cell replacement applications to the iPSC assisted bioengineering of body organs for personalized autologous body organ transplant. Recent work has demonstrated that the generation of organs from iPSCs is a future possibility. The development of embryonic-like organ structures bioengineered from iPSCs has been achieved, such as an early brain structure (cerebral organoids), bone, optic vesicle-like structures (eye), cardiac muscle tissue (heart), primitive pancreas islet cells, a tooth-like structure (teeth), and functional liver buds (liver). Thus, iPSC technology offers, in the future, the powerful and unique possibility to make body organs for transplantation removing the need for organ donation and immune suppressing drugs. Whilst it is clear that iPSCs are rapidly becoming the lead cell type for research into cell replacement therapy and body organ transplantation strategies in humans, it is not known whether (1) such transplants will stimulate host immune responses; and (2) whether this technology will be capable of the bioengineering of a complete and fully functional human organ. This review will not focus on reprogramming to iPSCs, of which a plethora of reviews can be found, but instead focus on the latest developments in direct reprogramming of cells, the bioengineering of body organs from iPSCs, and an analysis of the immune response induced by iPSC-derived cells and tissues.

## 1. Direct Reprogramming

Transdifferentiation, direct reprogramming or direct lineage reprogramming are the three different terms used for describing when the overexpression of certain factors makes a fully differentiated cell change its transcriptional and protein profile to directly convert it from one cell type into another without intermediate progenitor stages [1]. The historical origin of cell transdifferentiation has been elegantly reviewed before, clearly setting the stage for the future development and advancement of the field [2]. Many cell types have been made using direct reprogramming methodology as described below.

### 1.1. Myocytes and Cardiomyocytes

The first report of direct reprogramming or transdifferentiation was that describing the conversion of fibroblasts, chondrocytes, and retinal epithelium into skeletal muscle by the transfection of the transcription factor MyoD [3]. The transdifferentiation into cardiomyocytes has not proved so simple as a master regulator gene for cardiomyocytes, similar to MyoD for skeletal muscle determination, has not been found. The group of Srivastava designed a screening strategy in which a group of 14 candidate genes were tested in different combinations for the cardiomyocyte induction capacity [4]. After fine-tuning to find the best combination with the minimal number of genes, three factors, Gata4, Mef2c, and Tbx5, were identified that together induce direct reprogramming of source cells into cardiomyocytes. The direct reprogramming was successful from both dermal and cardiac fibroblasts and the resulting cardiomyocytes had features similar to neonatal cardiomyocytes including contractility. This strategy has several advantages compared to deriving cardiomyocytes from iPSCs, including a greater efficiency and speed in cardiomyocyte generation.

### 1.2. Central Nervous Systems Cells

The demonstration of *in vitro* direct reprogramming to neurons was first reported in 2002 when the conversion of astrocytes into neurons, by over-expression of Pax6 was described [5]. Because astrocytes share a common cell lineage to neurons, they require minimal manipulation to directly reprogram them to neurons and may not be the most feasible source of starting cells for transdifferentiation. More accessible cell types for direct reprogramming to neurons include; (i) fibroblasts by direct reprogrammed using Brn2, Ascl1, and Myt1l (BAM) [6]; (ii) hepatocytes using also BAM [7]; (iii) pericytes using Sox2 and Mash1 [8]; and cord blood using Sox2 and c-Myc [9].

Furthermore, fibroblasts can be directly lineage-reprogrammed into spinal motor neurons using Ascl1, Brn2, Myt1l, Lhx3, Hb9, Isl1, and Ngn2 [10]. Of particular interest is the ability to convert one neuronal subtype into another, namely embryonic and early postnatal callosal projection neurons into corticofugal projection neurons by overexpression of Fezf2 [11], indicating that there is a period after post-mitotic development when neurons can change their subtype. The demonstration that this is a stable conversion, at least during this post-mitotic period, remains to be done.

Current methodologies employed in direct reprogramming carry a number of concerns when considered for clinical application. Formerly, all transdifferentiation strategies had been achieved using doxycycline-inducible lentiviral vectors. Fears of genotoxic integration and tumorigenicity associated with this method have been voiced. Testing of new non-viral methods for converting fibroblasts into neurons, by using plasmids as a gene carriers coding for BAM [12], by microRNA mediated conversion [13] and by using chemical compounds alone [14], have all been tried. These methods begin to pave the way for future clinical application and further work in this direction is warranted.

To date, it has been shown *in vitro* that neurons made from either mouse or human cells by direct reprogramming methods are electrophysiologically active, form synapses *in vitro* and express markers of post-mitotic neurons. There exists evidence of induced-dopaminergic neurons partially integrating with local neuronal circuitry after ectopic transplantation in mouse striatum, suggesting that direct reprogramming methods can make functional neurons that integrate successfully [15].

The challenge remains to demonstrate direct reprogramming *in vivo* to generate human neurons, as been demonstrated in mice, and, thus, circumvent the need for cell transplants [11,16,17]. Moreover, future work to achieve the production of different classes of neurons, that are specifically lost in distinct neurodegenerative disorders, is warranted.

### 1.3. Immune B Cells into Macrophages and Treatment of Cancer

A leading laboratory in the field of direct reprogramming of cells, has transdifferentiated a B lymphocyte cell line into macrophage-like cells at 100% efficiency, within two to three days, using an estradiol-inducible form of C/EBPalpha [2,18]. They demonstrated that the reprogrammed cells are larger, contain altered organelle and cytoskeletal structures, are phagocytic, and exhibit an inflammatory response. They conclude that the robustness and speed of their system make it a versatile tool to study biochemical and biological aspects of lineage reprogramming [18]. Interestingly, the same group has taken this finding further and demonstrated transdifferentiation of leukemia cell lines into macrophages, thus, impairing their tumorigenicity [19]. This work leads to the exciting idea of using cellular transdifferentiation as a method to treat cancer [19].

### 1.4. Hepatocytes and Pancreatic β-Cells

Hepatocytes and pancreatic islet β-cells are two endoderm-derived cell types that are the subject of much attention due to their indispensable physiological functions and their association with various diseases. The adult β cells have very limited regenerative ability, which is insufficient to compensate for cell loss and is thus the cause of many diseases, including diabetes. For this reason, there has been a large effort to find new sources of β cells and other pancreatic cells. Until recently, efficient treatments for restoring the cellular functions of these cell types were unknown. Clinical studies have now demonstrated that cell transplantation-based therapy can support and restore functions of failed liver and pancreatic islets. Recent advances in hepatocyte and β cell transdifferentiation have provided valuable insights into how to regenerate and restore the normal function of the liver and the pancreas [20].

It has been reported that mouse embryonic and adult fibroblasts can be directly reprogrammed into functional hepatocyte-like cells using a combination of endodermal and hepatic transcription factors [21,22]. In both reports, hepatocyte-like cells exhibited typical hepatocyte morphology, gene expression and protein secretions. These cells may resemble an immature stage of hepatocyte differentiation. However, similar human hepatocyte-like cells have yet to be generated. Mesenchymal stem cells (MSC) are an alternative source of hepatic cells. The hepatocyte-like cells transdifferentiated from MSC can engraft into the parenchyma of the liver and at least partially restore liver functions in injury models *in vivo* [23]. Melton and his colleagues reported that re-activation of three transcription factors (Ngn3, Pdx1, and Mafa) *in vivo* was able to reprogram pancreatic exocrine cells into endocrine β cells in adult mice. They utilized a cell tracking system and demonstrated that overexpression of the three key pancreatic factors converted acinar cells to insulin-producing β cells. Moreover, these β cells rescued mice from streptozotocin-induced diabetes, demonstrating a clear functional capacity of the cells [24].

Another source of new pancreatic cells is the inter-endocrine transition in pancreatic islets. Recent work has demonstrated that new β cells arose from healthy α cells in islets through transdifferentiation, which is induced by fatal β-cells ablation, as in diabetes [25]. Their results argue that a deep lesion (total or near-total β-cell ablation) causes the release of some form of signal that allows prolonged and substantial β-cell regeneration. Such inter-endocrine spontaneous adult cell conversion could be harnessed towards methods of producing β-cells for diabetes therapies, either in differentiation settings *in vitro* or induced regeneration [25].

As liver and pancreas arise from the same bi-potential precursors in the anterior endoderm, it is reasonable to speculate that two closely related tissues may be inter-convertible. For example, the glucocorticoid dexamethasone can efficiently induce rat pancreatic exocrine cells into hepatocytes [26], and there have been reports of hepatocyte transdifferentiation of hepatocytes into pancreatic β cells involving several key pancreatic factors [27,28]. These encouraging recent advance in liver and pancreatic cell transdifferentiation should promote continued development of cell replacement techniques for the future treatment of many liver and pancreas related diseases.

### 1.5. Retinal Pigment Epithelium

It has recently been described the conversion of human foreskin fibroblast into retinal pigment epithelium (RPE)-like cells by defined factors [29]. They developed an RPE-specific Best1::GFP reporter, which could faithfully represent human RPE lineage commitment during human embryonic stem cell differentiation. Using this reporter system, they showed that a defined set of transcription factors can reprogram human fibroblasts into Best1:GFP+ve colonies. They demonstrated that these Best1:GFP+ve cells formed pigmented monolayer epithelium exhibiting molecular features of RPE lineage.

In the initial experiment, six RPE developmental transcription factors; *Rax*, *Crx*, *Pax6*, *Mitf*, *Otx2*, and *Nrl* were used [29]. Despite transfection of these factors through retroviruses, pGZ-BEST1-GFP through lentivirus and culturing on Matrigel-coated plates and in hESC culture medium to prime RPE lineage conversion, there was no morphological change and no Best1:GFP+ve cells. In the next experiment, epigenetic “plasticity” was increased with the addition of *cMyc* and *Klf4* factors. The efficiency of RPE induction was altered by removal of certain combination of the six transcription factors. It was found that *cMyc*, *Mitf*, *Otx2*, *Rax*, and *Crx* were critical for reprogramming of human fibroblasts into Best1::GFP+ cells, while *Klf4*, *Nrl*, and *Pax6*, individually, were not as important and could potentially be omitted or replaced by other factors without major impact of induced RPE phenotype at day 35 post-transduction [29]. In addition, they observed further maturation of the induced RPE-like cells when they were maintained in medium containing retinoic acid and sonic hedgehog after day 21 [29]. This offers hope that there may be a safer combination of small molecules and transcription factors that can be used to induce RPE lineage from somatic cells for therapeutic application. However, the compulsory requirement of the *cMyc* oncogene and *Klf4* to the transcription factor cocktail limits the clinical utility of this approach.

### 1.6. In Vivo Transdifferentiation

*In vivo* direct reprogramming strategies involve cell transdifferentiation conducted not *in vitro* but in an adult organ of a living organism. One of the first examples of *in vivo* direct reprogramming (transdifferentiation) was in mice by targeting differentiated pancreatic exocrine cells and making cells that closely-resembled β cells by the expression of Ngn3, Pdx1, and Mafa [24]. The first insulin positive cells appeared as early as day three after gene induction, however, the induced β-cells did not organize into islet structures but remained as single cells or small clusters. More recently, hESCs were differentiated *in vitro* into pancreatic progenitors that were engrafted into mice, they matured *in vivo* into insulin producing pancreatic endocrine cells and achieved normoglycemia 30 weeks after transplantation [30]. The field of *in vivo* direct reprogramming offers advantages over *in vitro* transdifferentiation prior to delivery of cells, however, this technology still requires a lot of work to make functionally safe cells. As an alternative approach to *in vivo* direct reprogramming, the bioengineering of organs from iPSCs may become an easier and quicker approach towards future tissue regeneration strategies.

## 2. iPSCs and Bioengineering Tissues

There have been many attempts to bioengineer different organs from iPSCs such as brain, liver, heart, and other tissues listed below. In general, the successful creation of tissues and organs from iPSCs requires:

1. An appropriate mixture of cells to recapitulate cell-cell interaction during organ development. This can be achieved either by differentiating the cells to early progenitors or by mixing them with other cell types or tissues.
2. Providing a 3D scaffold or giving the right conditions for assembly in 3D.
3. Providing the right extracellular matrix that resembles that of embryonic organogenesis.

### 2.1. Heart

The ability to generate functional cardiac tissue by *in vitro* tissue engineering with donor cardiomyocytes has been known for more than ten years [31,32,33,34] but more recent developments include *ex vivo* [35] and *in vivo* [36] methods, which also provide the vascular and fibrous elements of cardiac tissue. Cell sheet techniques allowed for iPSC-derived cardiomyocytes to be delivered to the porcine heart demonstrating acute benefits [37] and contractile and vascularized human cardiac organoids have also been created from iPSCs [38,39], which provide longer-term survival and contractility. However the ability to create whole functional hearts by means of tissue bioengineering has proven elusive. The closest result to this complex whole organ bioengineering task has been engineered heart tissue obtained by using human iPSC-derived from multipotential cardiovascular progenitors (MCP) implanted into a decellularized donor mouse heart [40]. In contrast to previous work that used donor cardiomyocytes, MCP—the earliest cardiac progenitors in heart development—gave rise to cardiomyocytes, smooth muscle cells and endothelial cells. The decellularized heart provided a 3D architecture and the complex natural extracellular matrix, which promoted cardiomyocyte proliferation, differentiation and myofilament formation. This use of native cardiac scaffold also avoided the biocompatibility problems of some artificial scaffold materials. The resulting heart tissues presented the requisite features of rhythmic mechanical force generation, electrophysiological characteristics and response to drugs that make it a valuable model for the study of heart development and drug screening. Nevertheless, several improvements still have to be made to enable its use in regenerative medicine: heart fibroblasts are missing, since they do not derive from MCP and a functional cardiac conduction system has not been demonstrated. As a result, the mechanical force generated is insufficient and these model organs lack a coordinated electrical propagation necessary for synchronization of the tissues.

### 2.2. Pancreas

Although very much in the early stages of development as a complete tissue, functional islets of langerhans have also been created *in vitro* from iPSCs [41]. A protocol has been developed to differentiate iPSCs into glucose-responsive functional islets, with a 3D structure similar to adult pancreatic islets and that secreted insulin and improved blood glucose levels in hyperglycemic mice. The protocol involved a two-step cell culture method; first the differentiation of iPSCs to immature pancreatic cells and then second, culture in specific conditions that allow islet formation. The islets exhibited distinct 3D structural features similar to adult pancreatic islets and secreted insulin in response to glucose concentrations. Mice transplanted with the iPSC-derived islets normalized their blood glucose levels after nine days [41].

### 2.3. Brain

Cerebral organoids have been made by culturing in a 3D system the neuroectoderm derived from human iPSC [42]. First, iPSCs were differentiated into embryoid bodies and from those neuroectoderm was derived. Neuroectodermal tissue was cultured in a 3D scaffold system of matrigel droplets and then transferred to a spinning bioreactor. The resulting three-dimensional tissue presented heterogeneous regions similar to human brain that were discrete but interdependent. These cerebral organoids, which the authors do not intend to use for regenerative purposes, recapitulated features of human cortical development. As mice and human brains have highly complex and integrated structures and developments, these organoids could be good candidates for the study of human brain development and modeling of brain disorders such as microencephaly.

### 2.4. Liver

A recent and exciting development in the field of bioengineering organs has been the creation of a liver bud which when transplanted into mice was able to rescue drug-induced liver failure [43]. In this case the researcher co-cultured iPSCs differentiated to hepatic endoderm with human mesenchymal stem cells and human umbilical endothelial cells. Co-cultured cells, when plated in a bi-dimensional matrigel layer self-organized into a three-dimensional system that the authors termed iPSC-derived liver buds. These liver buds were able to produce liver specific proteins such as albumin and were able to metabolize drugs ketoprofen and debrisoquine. Notably, the presence of human umbilical endothelial cells in the starting cell mixture provided the iPSC-derived liver buds with vessels that, when implanted in the mice, connected with the host vessels within 48 h. This vascular system, together with the 3D structure seems to be the key for successful engraftment and maturation.

### 2.5. Eye

iPSCs have been used to generate optic vesicle-like structures that generated retinal cell types suitable for *in vitro* studies and disease modeling [44]. The authors differentiated iPSCs into 3D optical vesicles with the capacity of self-assemble into rudimentary neuroretinal structures and which expressed markers of intercellular communication. The use of retinal pigmented epithelial (RPE) cells to treat eye disease is currently being evaluated in clinical trials, leading the way for future research to develop the bio-engineered eye structures.

### 2.6. Teeth

The generation of complex tooth-like structures has been recently created from human iPSCs [45]. The authors developed a bioengineering protocol that combines iPSC-derived dental epithelial sheets with embryonic dental mesenchyme. The epithelial sheets gave raise to ameoblasts that produced the enamel component of the tooth and the embryonic dental mesenchyme produced the dentin-pulp complex and peridontium, as well as provided odontogenic signals to the epithelial sheets. The formed regenerative teeth were very similar in structure and mineral content to normal teeth and presented comparable hardness. As the sources of endogenous dental epithelial cells are scarce, iPSCs can provide a promising source of dental epithelial seed cells for use in tooth tissue engineering.

### 2.7. Bone

The bone tissue-engineering field has also made use of iPSC technology to create functional bone substitutes [46]. To make bone, iPSCs were differentiated into mesenchymal progenitors that were subsequently grown in an osteoconductive scaffold-perfusion bioreactor. The dense bone-like structure that formed inside the scaffold presented a mature bone molecular pattern. When implanted into mice, the engineered bone-tissue was stable over the 12 weeks of the study, without differentiation into other lineages, displayed vascular ingrowth and connective tissue development, and presented signs of initiation of scaffold resorption.

### 2.8. Gut

3D gut organoids have also been created from iPSC [47]. A protocol has been developed in which iPSCs were differentiated into intestinal tissue following a sequential protocol mimicking embryonic development. Spontaneous three-dimensional spheroids formed that were transferred to three-dimensional culture systems known to promote intestinal formation. The resulting intestinal tissue presented a cellular composition similar to that of foetal intestine and also presented absorptive and secretory functions. However these gut organoids have not been tested for engraftment and function *in vivo*.

## 3. Immune Response of iPSC Derived Cells

As iPSC technology is advancing to become a future tool for clinical therapy, concerns about the susceptibility to immune rejection of iPSC grafts are increasing. As data accumulates, it has become apparent that iPSCs may not be as immune-privileged as initially thought [48]. Despite the sourcing of cells for iPSC treatment from the recipients, therefore overcoming HLA incompatibility, there remains the possibility that the reprogramming process itself might render the grafts or cells immunogenic [48]. Moreover, potential sources of innate proinflammatory “danger” signals that can lead to immune activation may be provided through the use of retroviral or episomal methods to make iPSC. Consistent with this, it has recently been demonstrated in mice that autologous transplantation of iPSCs, generated by episomal or retroviral vectors, elicited anti-graft T cell responses potent enough to prevent the formation of teratomas [49]. Interestingly, this was not observed with embryonic stem cells (ESC), where autologous transplantation in a syngeneic recipient did not elicit an immune response, suggesting the method to reprogram cells to the pluripotent state itself influences the ontogeny of immune responses within the host. Furthermore, the authors also found evidence of abnormal gene expression in some cells differentiated from iPSCs that were able to induce T-cell-dependent immune response in syngeneic recipients [49].

Recently, mouse iPSCs generated by lentivirus vectors or episomal vectors have been differentiated into representative cell types of the three germs layers and examined their immunogenicity *in vitro* and *in vivo* by transplantation into syngeneic recipients [50]. They found that differentiated cells derived from syngeneic iPSCs were not rejected after transplantation [50]. Moreover, model transplantation experiments were performed using various iPSC-derived differentiated tissues and immune-mediated rejections have not been observed. In further studies no differences in immunogenicity or transplantation success were found between differentiated skin and bone marrow tissue derived from integration-free mouse iPSCs (generated by episomal vectors) compared to that of ESC-derived tissue. This study did not observe any differences between the two groups in regards to the rate of transplantation success [51].

A very recent advance in this field has been a description of the induction of dopaminergic (DA) neurons from nonhuman primate iPSC by directed differentiation *in vitro*, and the comparison of the autologous and allogeneic transplantation into the brains of nonhuman primates [52]. Autologous transplantation of the iPSC-derived cells generated a minimal immune response compared with allografts in nonhuman primate brains in the absence of immunosupression. This data also entertains the idea that immunosupression is not necessary for autologous transplantation of iPSC-derived neural cells into brain. Moreover, they compared the immunogenicity of iPSCs generated with retroviral vectors *versus* episomal vectors. They detected that autologous grafts derived from iPSCs generated by retroviral vectors were infiltrated by a large number of IBA1^+^ and CD45^+^ microglia in comparison with those generated by episomal vectors, probably due to the residual expression of transgenes. This work suggests that residual transgenes can be immunogenic, for this reason it is crucial to use integration-free iPSCs [52].

Despite these recent advances, research to date has primarily focused on T cell mediated responses directed against stem grafts. However, many questions remain poorly understood, for example the role of NK cell mediated rejection in iPSC based transplantation technology. This seems to be especially of interest as the HLA class I and II expression on ESCs has been shown to vary depending on their maturation status [53]. Although a low expression of HLA has been thought to partially explain the reduced immunogenicity of ESCs, this reduced expression of HLA may in turn render these cells more vulnerable to NK cell mediated killing.

In summary, there is now encouraging evidence that grafts from terminally differentiated cells derived from syngeneic iPSCs can circumvent acute rejection in animal models. However, these studies also highlight the “devil in the detail” and show that even subtle differences in the generation of iPSCs may render them more or less immunogenic. Consequently, the future of the study of immunity against iPSCs is complicated by the simple truth that iPSCs are dynamic antigenic targets and that the immune response elicited by those cells will likely differ based on their generation, differentiation, age, and survival *in vivo*.

## 4. Conclusions

Both methods of direct reprogramming to desired cells types or full reprogramming to iPSCs and then differentiating to cells for replacement therapy have pros and cons for future clinical application. The length of time required for generation and differentiation of iPSCs into the desired cell type is the main handicap of iPSCs in comparison to directly reprogrammed cells. In addition differentiation of iPSCs to some cell types have not been achieved, for example, blood and functional sperm, with cells remaining in undifferentiated embryological cell states. Moreover, direct reprogramming avoids going through the big hurdle of the transient tumorigenic state of iPSCs. For this reason, direct reprogrammed cells could be a better choice in the clinical application for human cell replacement therapy for neurodegenerative disorders like Parkinson’s disease, Huntington’s disease, and amyotrophic lateral sclerosis.

However, directly reprogrammed cells are also susceptible to chromosomal aberrations [54,55] and all methods for direct reprogramming of cells to date have used viral transduction methods to insert genes into the genome of cells. Another con for direct reprogramming is that some cells have been shown to maintain an epigenetic memory of their cell of origin, like neurons, questioning their stability over time [56]. Moreover, with direct reprogramming the intermediate cell steps that form during the process are difficult to identify because the molecular markers they express remain unknown [1]. Therefore, iPSCs may be the cell choice for future regenerative medicine applications, given that they are well defined, characterized, and that their proliferative capacity is an advantage over post mitotically inactive direct reprogrammed cells.

Perhaps one of the major advantages of iPSCs will be for engineering of human organs for transplantation. The co-culturing of more than one cell type has proven successful for making a functional liver bud and although iPSCs were used in this study not all the cells used in the mix were derived from iPSCs [43]. Combining two or more cell types all derived from iPSCs to make complex organs like the liver, brain or heart may be needed in the near future. If this is possible, the powerful ability to efficiently produce pluripotent cells to seed the growth of autologous body organs for transplant medicine may be achievable in the near future.
